# Unveiling stages of change among medical inpatients with an increased-risk alcohol consumption—a cross-sectional study

**DOI:** 10.1093/alcalc/agaf067

**Published:** 2025-11-22

**Authors:** Maria Seferowicz, Anners Lerdal, Hilde Marie Erøy Edvardsen, Ragnhild Bergene Skråstad, Jørgen Valeur, Benedicte Marie Jørgenrud, Anna Armika Tussilago Nyman, Stig Tore Bogstrand

**Affiliations:** Department of Public Health Science and Interdisciplinary Health Sciences, Institute of Health and Society, Faculty of Medicine, University of Oslo, PO Box 1089 Blindern, 0317 Oslo, Norway; Medical Clinic Lovisenberg Diaconal Hospital, AS PO Box 4970 Nydalen 0440 Oslo, Norway; Department of Public Health Science and Interdisciplinary Health Sciences, Institute of Health and Society, Faculty of Medicine, University of Oslo, PO Box 1089 Blindern, 0317 Oslo, Norway; Research Department, Lovisenberg Diaconal Hospital, AS PO Box 4970 Nydalen 0440 Oslo, Norway; Section of Forensic Research, Department of Forensic Sciences Oslo University Hospital, PO Box 4950 Nydalen 0424 Oslo, Norway; Department of Clinical Pharmacology, St. Olavs Hospital, Trondheim University Hospital PO Box 3250 Torgarden, Norway; Department of Clinical and Molecular Medicine, NTNU, PO Box 89057491 Trondheim, Norway; Unger-Vetlesen Institute, Lovisenberg Diaconal Hospital, Lovisenberggata 21, 0456 Oslo, Norway; Institute of Clinical Medicine, University of Oslo, PO Box 1171 - Blindern, 0318 Oslo, Norway; Section of Forensic Research, Department of Forensic Sciences Oslo University Hospital, PO Box 4950 Nydalen 0424 Oslo, Norway; Section of Forensic Research, Department of Forensic Sciences Oslo University Hospital, PO Box 4950 Nydalen 0424 Oslo, Norway; Department of Public Health Science and Interdisciplinary Health Sciences, Institute of Health and Society, Faculty of Medicine, University of Oslo, PO Box 1089 Blindern, 0317 Oslo, Norway; Section of Forensic Research, Department of Forensic Sciences Oslo University Hospital, PO Box 4950 Nydalen 0424 Oslo, Norway

**Keywords:** alcohol consumption, AUDIT-C, phospatidylethanol, prevention paradox, increased-risk drinkers, stages of change

## Abstract

**Aims:**

Alcohol-related harm occurs at lower levels than previously recognized, warranting a need to identify mediators to reduce alcohol-associated risk in increased-risk drinkers. Stages of change (SOC) have been used to assess motivation for health behaviour alteration. The primary aim was to explore distribution on SOC among medical inpatients when comparing low-risk, increased-risk, and high-risk consumers as defined by Alcohol Use Disorder Identification Test-Consumption (AUDIT-C). Our secondary aim was to assess the distribution of SOC when consumption was stratified with a biomarker of alcohol use—phospatidylethanol 16:0/18:1.

**Methods:**

Cross-sectional study with three participating hospitals. Recruiting consecutive medical inpatients ≥18 years with regular alcohol consumption as screened by score ≥2 on the first question in AUDIT-C (*N* = 888). AUDIT-C score and SOC were assessed by questionnaires, and phospatidylethanol concentration in a blood sample. Odds ratios and the 95% confidence intervals were calculated through a univariate logistic regression analysis for each variable, and multivariable logistic regression models were then fit to calculate the adjusted odds ratio and 95% confidence interval.

**Results:**

Distributions of SOC differed between the three risk-groups. Distribution of SOC was comparable whether assessed by phospatidylethanol or AUDIT-C.

**Conclusions:**

Increased-risk consumers constitute the majority of those in action—the only stage associated with consecutive reduction in drinking. Potentially, these results can aid in reducing perceived barriers among health care professionals in screening and offering health advice to those with increased-risk consumption and inform further research on mediators in this subgroup.

## Teaser

**Short Summary:** Alcohol-related harm occurs at lower levels than previously recognized. This study investigated the distribution of groups with low, increased, and high-risk consumption in stages of change. Increased-risk consumers constitute the majority of those in action—the stage associated with consecutive reduction in drinking.

## Introduction

Alcohol use disorders (AUD) are highly prevalent—affecting 8.6% of men and 1.6% of women worldwide (WHO 2019) with the sex gap converging in upper- and middle-income countries (Andreacchi  *et al.*  2024). It remains among the most undertreated health conditions (Carvalho  *et al.*  2019). There is no lowest level of safe alcohol intake (Anderson  *et al.*  2023), and there is an increasing international recognition that even alcohol consumption within previously recommended guidelines is associated with health risks (Connor and Hall 2018, Sherk  *et al.*  2020). AUD (APA 2022) is in the upper part of a spectrum that ranges from use of alcohol above recommended limits to alcohol dependency (Saitz 2005).

Updated guidelines advise on consumption below three drinks a week as the risk of several types of cancers increases when this threshold is exceeded, thus defining ≥3 drinks a week as increased-risk consumption (Paradis  *et al.*  2023)*.* Estimates indicate that up to 50% of Canadian drinkers surpassed this intake in 2023 (Stockwell and Zhao 2023). Norway is a Nordic country with about 5.5 million citizens. The average alcohol consumption of about 7.5 l of pure ethanol per year in adults is lower than most European countries (WHO 2019)—nonetheless, it has been estimated that around 40% of medical inpatients encounter levels of alcohol consumption that exceed the low-risk limit (Kabashi  *et al.*  2019). Alcohol intake has an impact on a number of conditions that affect medical inpatients including several types of cancers, tuberculosis, lower respiratory infections, liver cirrhosis, and a wide range of cardiovascular conditions, such as hypertensive and ischaemic heart disease, stroke, conduction disorders, and other dysrhythmias (Rehm  *et al.*  2010).

Addressing risky alcohol use in a hospital setting might be efficient as hospitalized patients may be more likely to see effects associated with their health and often have established trust with the medical staff attending them (Tezuka  *et al.*  2024), providing a golden window of opportunity to offer advice regarding the health impacts of their alcohol consumption (Holloway  *et al.*  2007).

Screening routines are recommended as most health care professionals have been shown to have limited ability to identify which patients have AUDs (Mitchell  *et al.*  2012). It is reasonable to assume that this is true also for patients with increased-risk consumption. The way healthcare professionals perceive alcohol-related norms may interfere with their clinical assessment and motivation to address the potential harm in this group of patients (Tjelta  *et al.*  2024). Conceivably due to a combination of social acceptability of consumption at increased-risk levels and a wish to ‘protect’ patients from the stigma believed to be associated with hazardous or harmful use (Grønkjær  *et al.*  2017).

Emphasizing the need for screening is also the prevention paradox stipulating that even though the individual risk is higher in high-risk drinkers, the majority of alcohol-related problems can be attributed to the group of increased-risk drinkers as they are more numerous (Kreitman 1986). This was further highlighted in an epidemiological modelling study designed to better understand which consumers experience the highest aggregated harms from alcohol. The findings suggested that distribution of the highest alcohol harm density is between 10–30 g/day for women and 10–50 g/day for men (Sherk  *et al.*  2024). Identifying patients that exceed the recommended lower drinking limit (i.e. increased-risk drinkers) and advising them on drink reduction may be of greater importance than previously emphasized. Particularly, as many increased-risk drinkers do not seem to have awareness of the harmful effects of alcohol on their health (Bowden  *et al.*  2014). This forms a need for further recognition of possible mediators in this large group.

Baseline motivation to change has been highlighted as a possible mediator for reduction in alcohol consumption (Leontieva  *et al.*  2005). The Stages of Change Questionnaire (Rollnick  *et al.*  1992) is based on the Transtheoretical Model by DiClemente & Prochaska. Higher baseline stages of change (SOC)-scores may have predicative value regarding consumption and alcohol-related outcomes (Heather  *et al.*  1993). Although subjected to criticism (West 2005), there is a large scope of research with regards to SOC/readiness to change (RTC) and alcohol consumption in different patient populations; college students (Merrill  *et al.*  2015), army recruits (Gaume  *et al.*  2013), patients in the emergency department (Field  *et al.*  2020), general public (Baumann  *et al.*  2015), medical outpatients (Matwin and Chang 2011), and medical inpatients (Bertholet  *et al.*  2009b). Despite recognized weaknesses, SOC have been commonly used to assess motivation to change drinking behaviour (Tan  *et al.*  2023), and although different models are in development they are not yet well established internationally (Richards 2023).

Past research assessing SOC/RTC has used cut-offs of ≥8 for Alcohol Use Disorder Identification Test (AUDIT) (Bertholet  *et al.*  2009a, Reed  *et al.*  2019, Field  *et al.*  2020). Increased-risk consumers in this study are likely to fall below these previous cut-off limits, as they are defined by an Alcohol Use Disorder Identification Test Consumption (AUDIT-C) score of 3–5 for women and 4–6 for men. Reasonably many will not reach a total AUDIT score of ≥8, as they are less probable to get high scores in the second part of the Alcohol Use Disorder Identification Test—Problematic use.

The primary aim of this study was to investigate research gaps in the group of increased-risk alcohol consumers and explore how SOC were distributed among patients denoted as low, increased, and high-risk defined by AUDIT-C score. The hypothesis was that those with increased-risk consumption would likely be in the middle between the two other groups of low-risk and high-risk. Nevertheless, as the public has limited knowledge about the hazards associated with increased-risk consumption (Bates  *et al.*  2018), a result close to those in low-risk was our alternative hypothesis.

As both the SOC and the AUDIT-C are based on self-report, the potential for social desirability bias is increased. Hence, we pursued the opportunity to include a secondary aim and explored the association between SOC and risk-levels when consumption was stratified by phospatidylethanol 16:0/18:1 (PEth), an objective and validated ethanol biomarker, as compared to self-reported alcohol use. To acknowledge the bidirectional relationship between increased alcohol consumption and mental distress (Tyssen  *et al.*  1998, Hassan 2017), we included Symptom checklist-5 (SCL-5)-score as a variable to exclude potential bias related to mental health.

## Materials and Methods

### Design, participants, and setting

This was a cross-sectional study designed to explore SOC in medical inpatients with regular alcohol consumption. The study was reported in accordance with the 22-item checklist for Strengthening the Reporting of Observational Studies in Epidemiology - the STROBE-guidelines for cross-sectional studies. Participants were recruited from the standard of care group (SC) in the larger multicentre AlcoTail study. AlcoTail was a longitudinal, experimental, pre-post-test, register-based study on the effects of screening and tailored intervention of harmful alcohol use among hospitalized medical patients in Norway. It contained a control group of 2000 patients (included 2021–2023) that received SC prior to the intervention and a comparison population of 2000 patients (included 2022–2025) after the intervention was established.

Recruitment and data collection for the SC were conducted at three hospitals:

- (i) *St Olavs hospital Trondheim University Hospital* STO in Trondheim, Norway’s third largest city with an average of 12,000 acute medical inpatient admissions a year. Recruitment time between February and September 2022.
- (ii) *Oslo University Ullevål hospital* OUS—in Oslo, the capital of Norway, with an average of 12,000 acute medical inpatient admissions a year. Recruitment time September–November 2021.
- (iii) *Lovisenberg Diaconal hospital* LDS—a district general inner-city hospital with an average of 6000 acute medical inpatient admissions per year. Recruitment time between October 2022 and April 2023.

To be included in the parent study (AlcoTail), patients had to be admitted to a medical ward in one of the partaking hospitals. Participants were recruited consecutively by dedicated study staff. Additional inclusion criteria were ≥18 years of age and ability to speak and read Norwegian to fully understand and be capable to sign the informed consent. Attending staff were consulted about any potential cognitive impairments and the patient’s ability to participate in the study before inclusion, and patients who were confused or in delirium were not approached. Participants were able to withdraw from the study at any point without restriction. Apart from AUDIT-C/AUDIT, no priming questions that potentially influenced SOC-score were part of the parent study.

**Figure 1:**
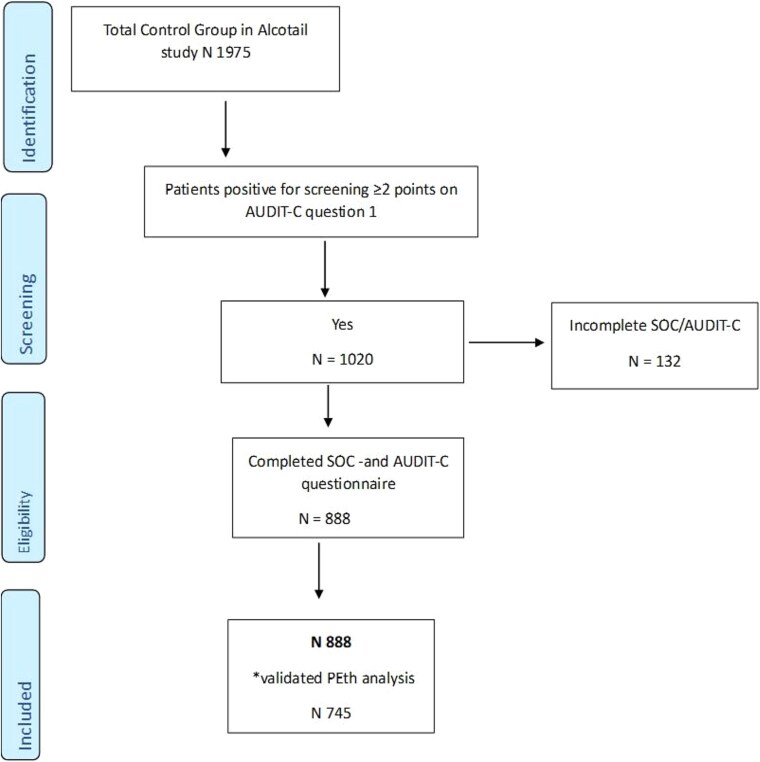
Prisma flow chart over patient number on identification, screening, eligibility, and final number of included patients in our study

To be identified for this study, participants had to be enrolled in SC in the Alco-Tail study. The participants were screened with regards to regular alcohol consumption and included if they had a score of ≥2 on the first AUDIT-C question. To be included, participants also had to fully complete the AUDIT-C and SOC-questionnaires as illustrated in Fig. 1.

This study was reviewed and approved by The Regional Committee for Ethics in Medical Research, South-Eastern Norway (REC# 200/31052) and authorized by the data protection officers at the three hospitals.

### Outcomes, measures, and data sources

Patients who consented to participation in the study completed a package of validated electronic questionnaires measuring alcohol consumption (AUDIT-C), SOC, and mental distress status (SCL-5). Designation to one of the three SOC was the primary outcome. A left-over blood sample was obtained from routine diagnostics drawn at the day of admission and was used to measure ethanol and the alcohol biomarker (PEth) which was applied to assess our secondary outcome.

#### Questionnaires

##### Alcohol Use Disorder Identification Test-Consumption

AUDIT-C was developed as a time-efficient screening to identify hazardous drinking habits in patients who have not yet experienced adverse effects from their alcohol consumption(Bush  *et al.*  1998). It is an abbreviated version of the full AUDIT and restricted to the first three questions about alcohol consumption. In this study, we adjusted for sex when assessing question three about heavy episode drinking (HED) >3/4 drinks per episode in women/men. Each item was answered on a five-point scale (zero to four points) with a total score (range zero to 12 points) designating the risk level.

Traditionally, AUDIT-C scores have been dichotomized to discriminate between presence and absence of at-risk drink behaviour. However, it is becoming more common to identify subgroups within the threshold of unhealthy alcohol used both in a clinical (Subhani  *et al.*  2024) and research aspect (Ryan  *et al.*  2022, Jack  *et al.*  2025). For the purpose of this study, we derived three drinking risk-levels and participants were designated to either low, increased, or high-risk according to the total AUDIT-C score. We did not include the frequency of HED as an additional criterion to define the level of risk drinking. The cut-off scores were sex-specific as women often tend to develop medical alcohol-related disease at lower thresholds of consumption than men (Fernández-Solà and Nicolás-Arfelis 2002). Women with a total AUDIT-C score ≤2 and men with a score of ≤3 were assigned to the low-risk group (Reinert and Allen 2007). Scores 3–5 for women and 4–6 for men constituted the increased-risk group (Wood  *et al.*  2024, Krist and Bradley 2025), as AUDIT-C scores between 7 and 12 are associated with high-risk drinking (Rubinsky  *et al.*  2013). We therefore decided on the high-risk drinking cut-off for men ≥7 and adjusted the cut-off for high-risk to ≥6 for women after performing sensitivity analyses (Table 4) with cut-off ≥7 and ≥5.

##### Stages of change

SOC-stage was determined by the Stages of Change Questionnaire—designed for brief opportunistic assessments in medical setting (Rollnick  *et al.*  1992). It consists of 12 questions with three four-item subscales assessing the patient’s beliefs about their current alcohol use and scoring them appropriately. We used a Norwegian translation of the questionnaires which gave points accordingly to the answer; disagree −1, unsure 0, or agree 1. Participants were assigned according to the quick method for allocation to the stage, with the highest score among the subscales represented the designated stage of change for the participant. SOC-stage as classified into precontemplation (PC), contemplation (C), and action (A). In case of equal scores, participants were manually allocated to the stage further along the continuum of change in accordance with the manual.

##### Symptom checklist-5

SCL-5 is a short-form screening tool validated for identifying and stratifying anxiety (two questions) and depression (three questions). The score is dichotomized with values >2 being an indicator of mental distress (Strand  *et al.*  2003).

#### Alcohol biomarkers

##### Phospatidylethanol 16:0/18:1

PEth is a specific biomarker for alcohol that reflects alcohol consumption over a period of 2–4 weeks before sampling. It is only formed in the presence of ethanol providing a high degree of specificity. PEth was analysed with validated ultra-performance liquid chromatography tandem mass spectrometry (UHPLC®-MSMS) methods at OUS and STO. Calibration range of the method was 0.030–4.00 μmol/l at both laboratories (Andreassen  *et al.*  2018).

PEth concentrations below 0.03 μmol/l (20 ng/ml) are compatible with abstinence or low-risk alcohol consumption. Concentrations higher than 0.3 μmol/l (200 ng/ml) are strongly suggestive of frequent excessive alcohol (i.e. high-risk) consumption, whereas concentrations between 0.03 and 0.3 μmol/l are indicating (i.e. increased-risk) alcohol consumption (Luginbühl  *et al.*  2022).

#### Demographic data

Demographic data were gathered from the patient record by dedicated research staff and included age (recorded into four groups; 18–40, 41–68, 68–80, >80 years), partnership status (single/widowed or married/living with partner), and employment status (student/employed or retired/disability benefits/unemployed).

### Data analysis strategy

Statistical analyses were performed using SPSS 29.00 (IBN Corp, Armonk, NY, USA). The sample size for this study was derived from the AlcoTail control group, where sample size was calculated based on the findings from a previous pilot study (Kabashi  *et al.*  2019).

Categorical variables were presented as numbers and percentages. To assess the normality of age as a continuous variable, we used the Kolmogorov–Smirnov test and reported the results as mean (SD). Independent sample t-test was performed to compare continuous variable (i.e. age), categorical data were compared with the Pearsons’s Chi-Square test. In case of missing data, the analysis was conducted with an adjusted number (*n*). Odds ratios (OR) and the 95% confidence intervals (CI) were calculated through a univariate logistic regression analysis for each of the variables as compared to AUDIT-C risk group (low-risk to increased-risk/low-risk to high-risk) and PEth risk group (low-risk to increased-risk/low-risk to high-risk). Multivariable logistic regression models were then fit to calculate the adjusted ORs and 95% CI. *P*-values below .05 were considered significant.

## Results

### Study sample

The AlcoTail control group had a total sample of 1975 patients from the three study sites (STO 497, OUS 981, and LDS 497 patients). A total of 1020 (52%) patients/participants had a score ≥2 on the first question in AUDIT-C. Eight hundred eighty-eight of these had completed the SOC and the full AUDIT-C questionnaire and were eligible for this study (Fig. 1).One hundred twenty-five patients were excluded due to incomplete SOC-questionnaire. The 125 patients had no differences in sex and PEth/AUDIT-C distribution. The only significant difference was in age-groups with more participants >68 years in the missing group. The 888 patients included in our study were slightly younger and with a higher number of male participants when compared to the total sample population—these findings are comparable to other populations of alcohol consumers (Abebe  *et al.*  2021). The mean age was 60.98 (SD 18.49) years in the study population and 65.12 (SD 18.58) years in the total sample with a two-sided *P* < .001. Male participants constituted 64% in our study population compared to 56% in the total sample population *P* < .001. Table 1 provides descriptive information of the sample as categorized by AUDIT-C score for the included participants. 

**Table 1:** Descriptive characteristics of the study population (n = 888).

AUDIT-C score women/men	Total (*N*)	Low risk <3/<4 (*N*)	Increased risk 3–5/4–6 (*N*)	High risk >5/>6 (*N*)	Missing *N*
Sex^*^					
Women	36% (316)	6% (51)	22% (193)	8% (72)	
Men	64% (572)	17% (154)	32% (287)	15% (131)	
	888	23% (205)	54% (480)	23% (203)	
Age^*^					
18–40 years	18% (161)	2% (14)	10% (91)	6% (56)	
41–67 years	37% (328)	6% (57)	21% (188)	9% (83)	
68–80 years	34% (304)	10% (89)	18% (160)	6% (55)	
>80 years	11% (94)	5% (45)	5% (40)	1% (9)	
	887	*205*	*479*	*203*	*1*
Marital status^*^					
Single/widowed	39% (332)	7% (59)	20% (167)	13% (106)	
Married/partner	61% (511)	17% (144)	34% (288)	9% (79)	
	843	*203*	*455*	*185*	*45*
Occupational status^*^					
Retired/disabled/unemployed	44% (366)	7% (56)	26% (215)	11% (95)	
Employed/student	56% (467)	17% (139)	28% (236)	11% (92)	
	833	*195*	*451*	*187*	*55*
Mental distress score^*^					
SCL 5 ≤ 2	86% (760)	22% (191)	47% (415)	17% (154)	
SCL 5 > 2	14% (122)	1% (12)	7% (62)	5% (48)	
	882	*203*	*477*	*202*	*6*
PEth^*^					
<0.03 μmol/l	35% (263)	15% (109)	19% (140)	2% (14)	
0.03–0.299 μmol/l	44% (328)	6% (46)	28% (205)	10% (77)	
≥0.3 μmol/l	21% (154)	1% (5)	8% (61)	12% (88)	
	745	*160*	*406*	*179*	*143*
SOC allocation^*^					
PC	71% (632)	22% (193)	40% (352)	10% (87)	
C	12% (103)	0% (2)	4% (36)	7% (65)	
A	17% (153)	1% (10)	10% (92)	6% (51)	

*p < 0.001 Italics number distribution in the three risk groups for each characteristic and the missing (showed in the last column).

### Stages of change distribution within risk-groups as assessed by **AUDIT-C and PEth**

**Figure 2:**
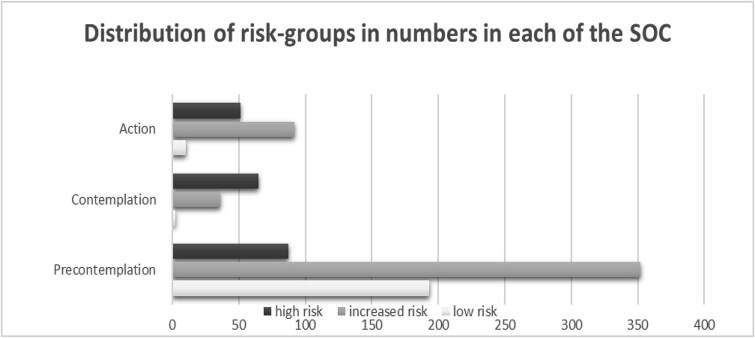
The distribution in numbers of participants in the three risk-groups low risk in light, increased risk in medium, and high risk in dark grey between the three SOC; PC, C, and A

**Figure 3:**
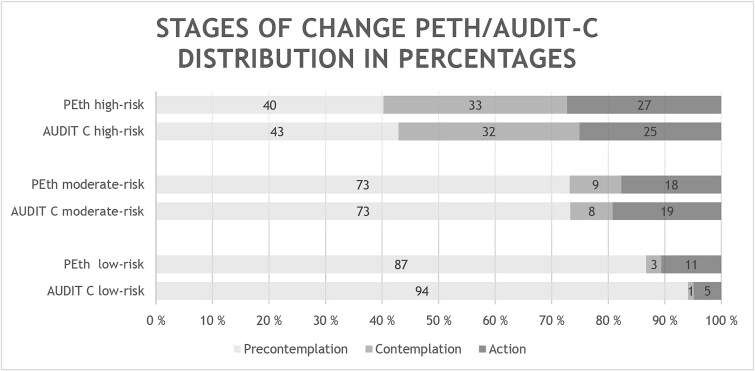
Comparing distribution of the three stages of change; PC—light, C- medium, and A- dark grey in percentages when comparing low, moderate, and high-risk consumption when stratified by self-reported questionnaire (AUDIT-C) on the inferior bar and when the consumption was stratified according to an objective and validated biomarker of alcohol use—PEth 16:0/18:1 on the superior bar

When assessed by AUDIT-C, 71% of the total study population were assigned to PC. The distribution varied between the three risk groups with 94% of the low-risk, 73% of the increased-risk, and 43% of the high-risk consumers in this stage. The distribution in C was quite different with 12% of the total study population in this stage, and the three risk groups distributed with <1% of the low-risk, 8% of the increased-risk, and high-risk 32%. Finally, A constituted for 17% of the total population 5% of the low-risk, 19% of the increased-risk, and 25% of high-risk consumers (Fig. 2 illustrating the distribution in numbers). The distribution was similar whether the risk assessment was done by self-reported screening or by the concentration of PEth (Fig. 3).

### Distribution of the three risk groups within each of the SOC

The group apportioned to the increased-risk consumers was twofold the size in numbers when compared to both the group of low-risk and high-risk consumers and constituted 54% of the total study population.

**Figure 4:**
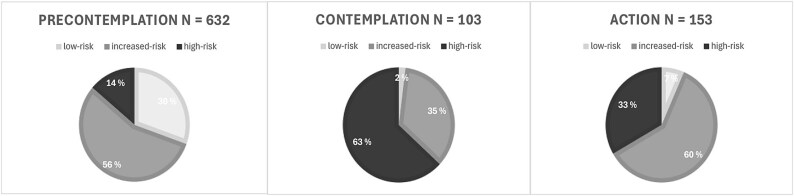
Pie-chart distribution percentage of low-risk (light grey), increased-risk (medium grey), and high-risk (dark grey) in the three stages of change; PC (n 632) C (n 103), and A (n 153)

The increased-risk consumers were in majority of the patients in PC 56%, and even more so in A 60%. The high-risk drinkers were in majority in C (Fig. 4).

### Primary outcome analysis

When consumption level was determined using AUDIT-C, the multivariable logistic regression analyses (Table 2) showed that the distributions of SOC stages were significantly different both for the increased-risk consumers (C OR = 9.63; CI = 2.2–42, *P* = .003 and A OR = 4.5; CI = 2.16–9.4, *P* < .001) and high-risk consumers (C OR = 60.5; CI = 13.8–270, *P* < .001 and A OR = 12.6; CI = 5.4–29, *P* < .001) when compared to the low-risk consumers.

**Table 2:** Multivariable logistic regressions association with AUDIT-C-stratified risk level.

Significant variables and CI-intervals AUDIT-C 3–5/4–6 increased-risk compared to AUDIT-C <3/<4 low-risk						
	Univariate analyses			Multivariate analyses		
			*P* > .25			
**Variables**	OR	95% CI	*P*	OR	95% CI	*P*
Sex Male 0/Female 1	2.03	1.4–2.9	<.001	2.09	1.38–3.16	<.001
^**^Occupation	0.44	0.3–0.64	<.001	0.86	0.45–1.66	.564
^**^Relationship status	0.70	0.49–1.01	.055	0.96	0.63–1.45	.829
^**^SCL-score	2.38	1.25–4.52	.008	1.4	0.69–2.85	.356
Age groups ^*^18–40						
41–67	0.5	0.3–0.96	.037	0.62	0.30–1.28	.199
68–80	0.28	0.15–0.51	<.001	0.4	0.16–1.00	.05
>80	0.14	0.07–0.28	<.001	0.19	0.07–0.53	.001
^*^SOC PC						
SOC C	9.87	2.35–41.4	.002	9.63	2.2–41.8	.003
SOC A	5.04	2.57–9.91	<.001	4.5	2.16–9.4	<.001
**Significant variables and CI-intervals AUDIT-C >5/>6 high-risk compared to AUDIT-C <3/<4 low-risk**						
**Variables**						
Sex Male 0/Female 1	1.66	1.08–2.54	.020	1.82	1.01–3.30	.047
^**^Occupation	0.39	0.26–0.60	<.001	0.98	0.42–2.28	.965
^**^Relationship status	0.31	0.20–0.47	<.001	0.43	0.24–0.76	.004
^**^SCL-score	4.96	2.55–9.67	<.001	1.40	0.54–3.56	.487
Age groups ^*^18–40						
41–67	0.36	0.19–0.72	.003	0.55	0.22–1.36	.193
68–80	0.15	0.08–0.30	<.001	0.25	0.08–0.79	.018
>80	0.05	0.02–0.13	<.001	0.07	0.02–0.28	<.001
^*^SOC PC						
SOC C	72.1	17.3–301	<.001	60.5	13.8–270	<.001
SOC A	11.31	5.49–23.3	<.001	12.6	5.4–29.4	<.001

^*^Indicator ^**^Dichotomic variable.

### Secondary outcome analysis

When risk-level of consumption was determined by use of an alcohol biomarker in the blood; PEth (Table 3), we found that the distribution of SOC stages was comparable to the self-reported classifications with significant differences both the increased-risk consumers (C OR = 3.77; CI = 1.6–8.9, *P* = .002 and A OR = 1.76; CI = 1–3, *P* = .037) and high-risk consumers (C OR = 20.9; CI = 8.7–51, *P* < .001 and A OR = 5.33; CI = 2.9–10, *P* < .001) when compared to the low-risk consumers.

**Table 3:** Multivariable logistic regressions association with PEth-stratified risk level.

Significant variables associated with PEth 0.03–0.299 increased risk compared to PEth < 0.03 low risk						
	Univariate analyses			Multivariate analyses		
			*P* > .25			
**Variables**	OR	95% CI	*P*	OR	95% CI	*P*
Sex Male 0/Female 1	0.82	0.59–1.15	.252	0.84	0.59–1.19	.326
^**^Occupation	0.73	0.52–1.02	.064	1.11	0.61–2.02	.729
^**^Relationship status	0.96	0.7–1.36	.834			
^**^SCL-score	0.98	0.59–1.61	.926			
Age groups ^*^18–40						
41–67	0.98	0.61–1.55	.971	1.07	0.64–1.8	.794
68–80	0.79	0.50–1.26	.326	0.93	0.43–1.99	.846
>80	0.35	0.19–0.64	<.001	0.44	0.17–0.98	.044
^*^SOC PC						
SOC C	4.07	1.75–9.45	.001	3.77	1.60–8.9	.002
SOC A	1.97	1.21–3.2	.006	1.76	1.04–3.0	.037
**Significant variables associated with PEth ≥ 0.3 high-risk compared to PEth < 0.03 low risk**						
**Variables**						
Sex Male 0/Female 1	0.51	0.33–0.78	.002	0.52	0.31–0.88	.016
^**^Occupation	1.02	0.67–1.55	.918			
^**^Relationship status	0.66	0.44–0.998	.049	0.77	0.46–1.28	.310
^**^SCL-score	2.07	1.22–3.52	.007	1.39	0.69–2.8	.361
Age groups ^*^18–40						
41–67	1.74	0.95–3.17	.071	2.69	1.24–5.83	.013
68–80	1.36	0.74–2.5	.321	2.31	1.04–5.83	.039
>80	0.31	0.12–0.78	.013	0.57	0.19–1.7	.318
^*^SOC PC						
SOC C	26.27	11.4–60.8	<.001	20.9	8.66–50.6	<.001
SOC A	5.52	3.17–9.6	<.001	5.33	2.89–9.9	<.001

^*^Indicator ^**^Dichotomic variable.

**Table 4:** Sensitivity analyses for AUDIT-C scores for women - cut off 5 and cut off 7

Significant variables associated with PEth 0.03–0.299 increased risk compared to PEth < 0.03 low risk						
	AUDIT C 5			AUDIT C 7		
			*P* > .25			
**Variables**	OR	95% CI	*P*	OR	95% CI	*P*
Sex Male 0/Female 1	1.62	1.1–2.5	.025	2.41	1.6–3.6	<.001
^**^Occupation	0.86	0.5–1.7	.653	0.86	0.5–1.7	.660
^**^Relationship status	0.95	0.6–1.5	.833	0.95	0.6–1.4	.815
^**^SCL-score	1.47	0.7–3	.292	1.37	0.7–2.8	.393
Age groups ^*^18–40						
41–67	0.74	0.4–1.6	.423	0.61	0.3–1.2	.175
68–80	0.5	0.2–1.2	.108	0.4	0.2–0.96	.041
>80	0.24	0.1–0.7	.006	0.18	0.1–0.5	<.001
^*^SOC PC						
SOC C	9.3	2.1–41	.003	10	2.3–43	.002
SOC A	4.5	2.2–9.4	<.001	4.6	2.2–9.6	<.001
**Significant variables associated with PEth ≥ 0.3 high-risk compared to PEth < 0.03 low risk**						
**Variables**						
Sex Male 0/Female 1	0.47	0.2–1.1	.067	3.76	2.2–6.4	<.001
^**^Occupation	1.08	0.4–2.8	.877	0.97	0.4–2.2	.933
^**^Relationship status	0.36	0.2–0.7	.002	0.47	0.3–0.8	.005
^**^SCL-score	1.88	0.54–3.56	.216	1.17	0.5–3	.747
Age groups ^*^18–40						
41–67	0.74	0.3–2	.558	0.42	0.2–0.99	.046
68–80	0.34	0.1–1.2	.1	0.24	0.08–0.7	.009
>80	0.1	0.02–0.5	.05	0.04	0.01–0.2	<.001
^*^SOC PC						
SOC C	69.6	16–308	<.001	63.6	14–292	<.001
SOC A	11.9	5–28	<.001	12.3	5.3–29	<.001

^*^Indicator ^**^Dichotomic variable. AUDIT-C scores: Men: low-risk <4 increased-risk 4–6 high-risk >6 Women: low-risk<3 increased-risk 4/(4-6) high-risk ≥5/ (≥7).

## Discussion

This study showed that there were significant differences in SOC-allocation among medical inpatients when classified into three groups of low, increased, or high-risk alcohol intake both by AUDIT-C and PEth. The distribution was similar to previous studies on non-treatment seeking medical patients with a majority in PC (Matwin and Chang 2011) and the SOC/RTC increasing with higher alcohol use severity.

Being classified into either of the two later stages (C, A) indicates that the patient could be considering or engaging in a change in behaviour (Heather  *et al.*  1993). More recent studies have implied that only A tends to be associated with a positive result on succeeding with consecutive reduction in alcohol consumption in patient populations both with and without intervention (follow-up time 3–12 months) (Reed  *et al.*  2005, Bertholet  *et al.*  2009a, Gaume  *et al.*  2013, Field  *et al.*  2020). Our findings indicate the highest fraction in A are those with increased-risk consumption. They make for 54% of the total population, but in A they are in majority with 60% and approx. one of five increased-risk consumers is in the action stage. This new knowledge could be of impact on previously perceived barriers for health professionals with regards to offering health advice on alcohol to this group of consumers (Grønkjær  *et al.*  2017, Tjelta  *et al.*  2024). With findings suggesting that a lot of the aggregated health harm is experienced within the increased-risk range (Sherk  *et al.*  2024) health promotion through offering patients personalized feedback about their risk may effectuate on an individual level with the prospect of risk reduction in diseases like cancer (Esser  *et al.*  2024). Further gains could potentiate on a population level when the theory of collectivity on drinking (Skog and Rossow 2006) is related for, as an reduction in alcohol use among the large population of increased-risk consumers may lead to a prospective decline in alcohol intake also among those with high-risk consumption.

As earlier research on mediator effects in SOC has been conducted in patients with a hazardous (AUDIT ≥8) alcohol consumption (Reed  *et al.*  2005, Bertholet  *et al.*  2009a), we do not yet know the predicative value of SOC in the subgroup of increased-risk consumers. Alcohol use severity is often proposed as a potential moderator for whether SOC mediate alcohol reduction and individuals with less severe drinking habits are thought to be more prone to respond to brief interventions (Moyer  *et al.*  2002). This informs further research to address knowledge gaps with regards to effects of screening and potential intervention in the increased-risk subpopulation.

Past research has found moderate to high correlations between the PEth value and AUDIT-C-scores (Jørgenrud  *et al.*  2021, Verheij  *et al.*  2022). To our knowledge, all previous SOC/RTC-studies have used self-report measures of alcohol consumption, while this study also included stratification of alcohol consumption by PEth. With regards to the secondary aim of our study, our findings exhibited similar distribution of the baseline RTC stages within the three risk groups when assessed by AUDIT-C questionnaire and compared to three risk groups as defined by levels of the PEth-biomarker.

While different assessment of alcohol risk-levels are becoming more common, the cut-offs both for AUDIT-C and PEth to differentiate between the groups of increased-risk and high-risk consumers are still not well established. Though not designed with this purpose—the findings of homogeneity within the groups as well as the heterogenicity between the three groups might be a support for the AUDIT-C cut-offs used in this study. As it becomes more apparent that considerable health hazards emerge already with a consumption ≥3 units per week, there will probably be a need to address risks at lower PEth-values than previously identified. Possibly, advice could be offered when PEth exceeds 0.03 μmol/l with regards to risk related to cancer and cardiovascular diseases (Hahn  *et al.*  2023), and the results in our study identify a difference in motivation to change below and above this threshold.

### Limitations

This study included 888 medical inpatients with a wide age range across three hospital sites in Norway, thereby providing ample statistical power, reducing potential bias, and increasing generalizability of the findings. Our study sample should be representative for Norwegian residents, but the inclusion criteria yielded recruitment bias as patients with insufficient understanding of the Norwegian language were excluded. This is a limitation, and supplementary research is necessitated to determine whether our results are reproducible internationally. An additional limitation was that the 132 patients who were excluded from the study due to deficient questionnaire completion (7 patients Audit-C/125 patients SOC) - could generate a potential selection bias towards participants with higher levels of motivation. A modified measure of the SOC-questionnaire with a three-point likert scale (as compared to the original five-point scale) was chosen to reduce the burden of assessment, but became a limitation when comparing the results to other studies and assessing a continuous SOC-score. Further, use of the quick method to allocate SOC does not acknowledge that the stages are not mutually exclusive and is also a limitation in this study. However, for the purpose of assessing the prevalence of SOC in this sample, it is probably sufficient (Forsberg  *et al.*  2004). Nonetheless, a single assessment of SOC will not be able to capture the complex process needed to implement change in alcohol consumption and constitutes a limitation when interpreting the implications of the findings.

## Conclusion

Our results found differences in the distribution of SOC between low, increased, and high-risk alcohol consumers. This observation was equivalent both when alcohol consumption was assessed with a self-report AUDIT-C questionnaire and with the ethanol biomarker PEth. Action stage has been associated with a consecutive reduced drinking pattern, and our study found that the increased-risk drinkers made up the majority in this stage. There are some limitations to this finding, as it remains to assess the mediator effect of SOC in the group of increased-risk drinkers. Nonetheless, the results can have implications for the perceived barriers among health professionals in screening to identify and offer health advice to raise awareness of potential health hazards caused by alcohol in this large group of consumers. We therefore believe our findings to lend further support to the endeavour of targeting prevention strategies on entire populations. Potential impacts can manifest on an individual level with risk reduction for diseases like cancer and cardiovascular disease, as well as on a population level. However, to assess the efficacy of such approach, further research with interventional studies in increased-risk alcohol consumers is required.

## Supplementary Material

STROBE_checklist_article_SOC_increased_risk_drinkers_agaf067

## Data Availability

Data underlying this article can be shared if permission is granted.

## References

[ref1] Abebe DS, Lien L, Bramness JG. Effects of age and gender on the relationship between alcohol use disorder and somatic diseases: a national register study in Norway. BMJ Open. 2021;11:e050608. 10.1136/bmjopen-2021-050608 PMC8587343 34758993

[ref2] Anderson BO, Berdzuli N, Ilbawi A. et al. Health and cancer risks associated with low levels of alcohol consumption. Lancet Public Health. 2023;8:e6–7. 10.1016/S2468-2667(22)00317-6 36603913 PMC9831798

[ref3] Andreacchi AT, Hobin E, Siddiqi A. et al. Age, period and cohort effects of heavy episodic drinking by sex/gender and socioeconomic position in Canada, 2000–2021. Addiction. 2024;119:2162–73. 10.1111/add.16641 39228260

[ref4] Andreassen TN, Havnen H, Spigset O. et al. High throughput UPLC®-MSMS method for the analysis of phosphatidylethanol (PEth) 16:0/18:1, a specific biomarker for alcohol consumption, in whole blood. J Anal Toxicol. 2018;42:33–41. 10.1093/jat/bkx075 28977407

[ref5] APA . Desk Reference to the Diagnostic Criteria from DSM-5-TR [TM]. 2022 Amer Psychiatric Pub Inc.

[ref6] Bates S, Holmes J, Gavens L. et al. Awareness of alcohol as a risk factor for cancer is associated with public support for alcohol policies. BMC Public Health. 2018;18:688. 10.1186/s12889-018-5581-8 29866082 PMC5987582

[ref7] Baumann S, Gaertner B, Schnuerer I. et al. The impact of a stage tailored intervention on alcohol use trajectories among those who do not intend to change. Drug Alcohol Depend. 2015;147:167–74. 10.1016/j.drugalcdep.2014.11.020 25500129

[ref8] Bertholet N, Cheng DM, Palfai TP. et al. Does readiness to change predict subsequent alcohol consumption in medical inpatients with unhealthy alcohol use? Addict Behav. 2009a;34:636–40. 10.1016/j.addbeh.2009.03.034 19428189 PMC2789443

[ref9] Bertholet N, Dukes K, Horton NJ. et al. Factor structure of the SOCRATES questionnaire in hospitalized medical patients. Addict Behav. 2009b;34:568–72. 10.1016/j.addbeh.2009.03.013 19395177 PMC2683890

[ref10] Bowden JA, Delfabbro P, Room R. et al. Alcohol consumption and NHMRC guidelines: has the message got out, are people conforming and are they aware that alcohol causes cancer? Aust N Z J Public Health. 2014;38:66–72. 10.1111/1753-6405.12159 24494949

[ref11] Bush K, Kivlahan DR, McDonell MB. et al. The AUDIT alcohol consumption questions (AUDIT-C): an effective brief screening test for problem drinking. Arch Intern Med. 1998;158:1789–95. 10.1001/archinte.158.16.1789 9738608

[ref12] Carvalho AF, Heilig M, Perez A. et al. Alcohol use disorders. Lancet. 2019;394:781–92. 10.1016/S0140-6736(19)31775-1 31478502

[ref13] Connor J, Hall W. Thresholds for safer alcohol use might need lowering. Lancet. 2018;391:1460–1. 10.1016/S0140-6736(18)30545-2 29676264

[ref14] Esser MB, Sherk A, Liu Y. et al. Reducing alcohol use to prevent cancer deaths: estimated effects among U.S. adults. Am J Prev Med. 2024;66:725–9. 10.1016/j.amepre.2023.12.003 38514233 PMC10963036

[ref15] Fernández-Solà J, Nicolás-Arfelis JM. Gender differences in alcoholic cardiomyopathy. J Gend Specif Med. 2002;5:41–7. 11859686

[ref16] Field CA, Richards DK, Castro Y. et al. The effects of a brief motivational intervention for alcohol use through stages of change among nontreatment seeking injured patients. Alcohol Clin Exp Res. 2020;44:2361–72. 10.1111/acer.14466 32981123 PMC7680429

[ref17] Forsberg L, Ekman S, Halldin J. et al. The readiness to change questionnaire: reliability and validity of a Swedish version and a comparison of scoring methods. Br J Health Psychol. 2004;9:335–46. 10.1348/1359107041557084 15296681

[ref18] Gaume J, Bertholet N, Daeppen JB. et al. The Change Questionnaire predicts change in hazardous tobacco and alcohol use. Addict Behav. 2013;38:2718–23. 10.1016/j.addbeh.2013.07.004 23934002

[ref19] Grønkjær M, Søndergaard LN, Klit MØ. et al. Alcohol screening in North Denmark Region hospitals: frequency of screening and experiences of health professionals. Nordisk Alkohol Nark. 2017;34:230–42. 10.1177/1455072517691057 32934487 PMC7450869

[ref20] Hahn JA, Fatch R, Barnett NP. et al. Phosphatidylethanol vs transdermal alcohol monitoring for detecting alcohol consumption among adults. JAMA Netw Open. 2023;6:e2333182. 10.1001/jamanetworkopen.2023.33182 37698861 PMC10498325

[ref21] Hassan AN . Patients with alcohol use disorder co-occurring with depression and anxiety symptoms: diagnostic and treatment initiation recommendations. J Clin Psychiatry. 2017;79:69–71. 10.4088/JCP.17ac11999 29244266

[ref22] Heather N, Rollnick S, Bell A. Predictive validity of the Readiness to Change Questionnaire. Addiction. 1993;88:1667–77. 10.1111/j.1360-0443.1993.tb02042.x 8130706

[ref23] Holloway AS, Watson HE, Arthur AJ. et al. The effect of brief interventions on alcohol consumption among heavy drinkers in a general hospital setting. Addiction. 2007;102:1762–70. 10.1111/j.1360-0443.2007.01968.x 17784901

[ref24] Jack HE, Berger DB, Bobb JF. et al. Association between change in alcohol use reported during routine healthcare screening and change in subsequent hospitalization: a retrospective cohort study. Addiction. 2025;120:884–94. 10.1111/add.16771 39868613 PMC12204387

[ref25] Jørgenrud B, Kabashi S, Nadezhdin A. et al. The association between the alcohol biomarker phosphatidylethanol (PEth) and self-reported alcohol consumption among Russian and Norwegian medical patients. Alcohol Alcohol. 2021;56:726–36. 10.1093/alcalc/agab013 33677484 PMC8557652

[ref26] Kabashi S, Vindenes V, Bryun EA. et al. Harmful alcohol use among acutely ill hospitalized medical patients in Oslo and Moscow: a cross-sectional study. Drug Alcohol Depend. 2019;204:107588. 10.1016/j.drugalcdep.2019.107588 31590131

[ref27] Kreitman N . Alcohol consumption and the preventive paradox★. Br J Addict. 1986;81:353–63. 10.1111/j.1360-0443.1986.tb00342.x 3461846

[ref28] Krist AH, Bradley KA. Addressing alcohol use. N Engl J Med. 2025;392:1721–31. 10.1056/NEJMcp2402121 40305713

[ref29] Leontieva L, Horn K, Haque A. et al. Readiness to change problematic drinking assessed in the emergency department as a predictor of change. J Crit Care. 2005;20:251–6. 10.1016/j.jcrc.2005.05.009 16253794

[ref30] Luginbühl M, Wurst FM, Stöth F. et al. Consensus for the use of the alcohol biomarker phosphatidylethanol (PEth) for the assessment of abstinence and alcohol consumption in clinical and forensic practice (2022 Consensus of Basel). Drug Test Anal. 2022;14:1800–2. 10.1002/dta.3340 35851997

[ref31] Matwin S, Chang G. Readiness to change and risk drinking women. J Subst Abuse Treat. 2011;40:230–40. 10.1016/j.jsat.2010.11.004 21193283 PMC3072060

[ref32] Merrill JE, Wardell JD, Read JP. Is readiness to change drinking related to reductions in alcohol use and consequences? A week-to-week analysis. J Stud Alcohol Drugs. 2015;76:790–8. 10.15288/jsad.2015.76.790 26402360 PMC4714829

[ref33] Mitchell AJ, Meader N, Bird V. et al. Clinical recognition and recording of alcohol disorders by clinicians in primary and secondary care: meta-analysis. Br J Psychiatry. 2012;201:93–100. 10.1192/bjp.bp.110.091199 22859576

[ref34] Moyer A, Finney JW, Swearingen CE. et al. Brief interventions for alcohol problems: a meta-analytic review of controlled investigations in treatment-seeking and non-treatment-seeking populations. Addiction. 2002;97:279–92. 10.1046/j.1360-0443.2002.00018.x 11964101

[ref35] Paradis C. et al. Low-Risk Alcohol Drinking Guidelines Scientific Expert Panels (2023) Canada’s guidance on alcohol and health: final report. Ottawa. Ont: Canadian Centre on Substance Use and Addiction.

[ref36] Reed DN Jr, Wolf B, Barber KR. et al. The stages of change questionnaire as a predictor of trauma patients most likely to decrease alcohol use. J Am Coll Surg. 2005;200:179–85. 10.1016/j.jamcollsurg.2004.10.020 15664091

[ref37] Reed MB, Woodruff SI, Holt M. et al. The relationship between self-efficacy, readiness to change, and AUDIT risk levels in a sample of active duty emergency department patients. Mil Psychol. 2019;31:187–99. 10.1080/08995605.2019.1579607

[ref38] Rehm J, Baliunas D, Borges GLG. et al. The relation between different dimensions of alcohol consumption and burden of disease: an overview. Addiction. 2010;105:817–43. 10.1111/j.1360-0443.2010.02899.x 20331573 PMC3306013

[ref39] Reinert DF, Allen JP. The alcohol use disorders identification test: an update of research findings. Alcohol Clin Exp Res. 2007;31:185–99. 10.1111/j.1530-0277.2006.00295.x 17250609

[ref40] Richards DK . Advancing theory of motivation to change alcohol use: a commentary on Tan *et al.* (2023). Alcohol Clin Exp Res (Hoboken). 2023;47:1833–5. 10.1111/acer.15171 37864531 PMC10605894

[ref41] Rollnick S, Heather N, Gold R. et al. Development of a short ‘readiness to change’ questionnaire for use in brief, opportunistic interventions among excessive drinkers. Br J Addict. 1992;87:743–54. 10.1111/j.1360-0443.1992.tb02720.x 1591525

[ref42] Rubinsky AD, Dawson DA, Williams EC. et al. AUDIT-C scores as a scaled marker of mean daily drinking, alcohol use disorder severity, and probability of alcohol dependence in a U.S. general population sample of drinkers. Alcohol Clin Exp Res. 2013;37:1380–90. 10.1111/acer.12092 23906469

[ref43] Ryan ED, Chang YM, Oliver M. et al. An Alcohol Symptom Checklist identifies high rates of alcohol use disorder in primary care patients who screen positive for depression and high-risk drinking. BMC Health Serv Res. 2022;22:1123. 10.1186/s12913-022-08408-1 36064354 PMC9446862

[ref44] Saitz R . Unhealthy alcohol use. N Engl J Med. 2005;352:596–607. 10.1056/NEJMcp042262 15703424

[ref45] Sherk A, Thomas G, Churchill S. et al. Does drinking within low-risk guidelines prevent harm? Implications for high-income countries using the international model of alcohol harms and policies. J Stud Alcohol Drugs. 2020;81:352–61. 10.15288/jsad.2020.81.352 32527387

[ref46] Sherk A, Churchill S, Cukier S. et al. Distributions of alcohol use and alcohol-caused death and disability in Canada: defining alcohol harm density functions and new perspectives on the prevention paradox. Addiction. 2024;119:696–705. 10.1111/add.16414 38237919

[ref47] Skog OJ, Rossow I. Flux and stability: individual fluctuations, regression towards the mean and collective changes in alcohol consumption. Addiction. 2006;101:959–70. 10.1111/j.1360-0443.2006.01492.x 16771888

[ref48] Stockwell T, Zhao J. Estimates of compliance with Canada's guidelines for low and moderate risk alcohol consumption: the importance of adjustment for underreporting in self-report surveys. Can J Public Health. 2023;114:967–72. 10.17269/s41997-023-00781-6 37213033 PMC10726685

[ref49] Strand BH, Dalgard OS, Tambs K. et al. Measuring the mental health status of the Norwegian population: a comparison of the instruments SCL-25, SCL-10, SCL-5 and MHI-5 (SF-36). Nord J Psychiatry. 2003;57:113–8. 10.1080/08039480310000932 12745773

[ref50] Subhani M, Nath DR, Talat U. et al. Screening for alcohol use disorder among hospitalised patients: learning from a retrospective cohort study in secondary care. J Clin Med. 2024;13:7617. 10.3390/jcm13247617 PMC11678479 39768540

[ref51] Tan Z, Tanner-Smith EE, Walters ST. et al. Do brief motivational interventions increase motivation for change in drinking among college students? A two-step meta-analysis of individual participant data. Alcohol Clin Exp Res (Hoboken). 2023;47:1433–46. 10.1111/acer.15126 37526588 PMC10692312

[ref52] Tezuka Y, So R, Fukuda T. The effectiveness of an ultra-brief intervention in 1 min for hazardous drinking in a general hospital setting: a quasi-randomized pilot trial. PCN Rep. 2024;3:e216. 10.1002/pcn5.216 38904063 PMC11187907

[ref53] Tjelta T, Bogstrand ST, Lerdal A. et al. Screening and following up harmful alcohol use “… is not necessarily your primary focus”: a qualitative study exploring health professionals’ experiences addressing harmful alcohol use in a Norwegian Hospital. J Multidiscip Healthc. 2024;17:5189–98. 10.2147/JMDH.S475750 39558926 PMC11571926

[ref54] Tyssen R, Vaglum P, Aasland OG. et al. Use of alcohol to cope with tension, and its relation to gender, years in medical school and hazardous drinking: a study of two nation-wide Norwegian samples of medical students. Addiction. 1998;93:1341–9. 10.1046/j.1360-0443.1998.93913415.x 9926540

[ref55] Verheij C, Haagsma JA, Koch BCP. et al. Screening for hazardous alcohol use in the emergency department: comparison of phosphatidylethanol with the alcohol use disorders identification test and the timeline follow-back. Alcohol Clin Exp Res. 2022;46:2225–35. 10.1111/acer.14958 36520053 PMC10107187

[ref56] West R . Time for a change: putting the Transtheoretical (stages of change) model to rest. Addiction. 2005;100:1036–9. 10.1111/j.1360-0443.2005.01139.x 16042624

[ref57] WHO, Hammer JH. *Global Status Report on Alcohol and Health 2018*. Geneva: World Health Organization; 2018. Licence: CC BY-NC-SA 3.0 IGO.

[ref58] Wood E, Pan J, Cui Z. et al. Does this patient have alcohol use disorder?: the rational clinical examination systematic review. JAMA. 2024;331:1215–24. 10.1001/jama.2024.3101 38592385

