# Supplementary material for: Unveiling stages of change among medical inpatients with an increased-risk alcohol consumption—a cross-sectional study
Source: Alcohol Alcohol. 2025 Nov 22;61(1):agaf067. doi: 10.1093/alcalc/agaf067 (PMC12640199; doi:10.1093/alcalc/agaf067)
Supplement: STROBE_checklist_article_SOC_increased_risk_drinkers_agaf067 [file strobe_checklist_article_soc_increased_risk_drinkers_agaf067.docx]

**SUPPLEMENTAL MATERIALS**

**Supplemental Table 1** – STROBE checklist

STROBE Statement—checklist of items that should be included in reports of observational studies

|  | Item No. | Recommendation | Page  No. | Relevant text from manuscript |
| --- | --- | --- | --- | --- |
| **Title and abstract** | 1 | (*a*) Indicate the study’s design with a commonly used term in the title or the abstract | 1 | “A *cross-sectional study*” (title) |
|  |  | (*b*) Provide in the abstract an informative and balanced summary of what was done and what was found | 2 | See abstract full text |
| Introduction | | | |  |
| Background/rationale | 2 | Explain the scientific background and rationale for the investigation being reported | 3-4 | See paragraphs 1-4 of the introduction |
| Objectives | 3 | State specific objectives, including any prespecified hypotheses | 5 | how SOC was distributed among patients  denoted as low, increased and high-risk  defined by AUDIT-C score. The  hypothesis was that those with  increased-risk consumption would likely  be in the middle between the two other  groups of low-risk and high-risk.  Secondary objective to explore the  association between SOC and  risk-levels when consumption was  stratified by Phospatidylethanol  16:0/18:1 PEth |
| Methods | | | |  |
| Study design | 4 | Present key elements of study design early in the paper | 6 | See “Design and settings” subsection  of the Methods |
| Setting | 5 | Describe the setting, locations, and relevant dates, including periods of recruitment, exposure, follow-up, and data collection | 6 | “Medical wards in three hospitals  in Norway – two in Oslo and one in  Trondheim. Data collection was  conducted in the period  September 2021- April 2023.” |
| Participants | 6 | (*a*) *Cohort study*—Give the eligibility criteria, and the sources and methods of selection of participants. Describe methods of follow-up  *Case-control study*—Give the eligibility criteria, and the sources and methods of case ascertainment and control selection. Give the rationale for the choice of cases and controls  ***Cross-sectional study***—Give the eligibility criteria, and the sources and methods of selection of participants | 6 | Admitted medical inpatients ≥18 years  in participating hospitals – as  further described under “participants”  subsection of Methods. |
|  |  | (*b*) *Cohort study*—For matched studies, give matching criteria and number of exposed and unexposed  *Case-control study*—For matched studies, give matching criteria and the number of controls per case | N/A | *Not matching study* |
| Variables | 7 | Clearly define all outcomes, exposures, predictors, potential confounders, and effect modifiers. Give diagnostic criteria, if applicable | 7-8 | Primary Outcome: classification to stages  of readiness to change  Secondary Outcome:  PEth-concentration  Potential confounders were assessed by  SCL-score, as well as demographic  data all described in the  “outcomes, measures and data sources”  subsection of Methods |
| Data sources/ measurement | 8* | For each variable of interest, give sources of data and details of methods of assessment (measurement). Describe comparability of assessment methods if there is more than one group | 8 | Data was sourced from self-reported  questionnaires for the Readiness of  Change stage, AUDIT-C score and  SCL-score. Ethanol and PEth  Concentration from blood sample  on admission. |
| Bias | 9 | Describe any efforts to address potential sources of bias | 7-8 | By ensuring a large study sample from  three different study sites and consecutive  recruiting we attempted to minimize  potential sources of bias. |
| Study size | 10 | Explain how the study size was arrived at | 9 | The participants in our study were  selected as a subpopulation of regular  alcohol consumers from the parent  AlcoTail study – the population size of  The parent study was calculated  based on findings from a pilot study. |

| Quantitative variables | | 11 | | Explain how quantitative variables were handled in the analyses. If applicable, describe which groupings were chosen and why | 9 | | We chose to define three groups  both with AUDIT-C and PEth  to identify those with low,  moderate and increased-risk |
| --- | --- | --- | --- | --- | --- | --- | --- |
| Statistical methods | | 12 | | (*a*) Describe all statistical methods, including those used to control for confounding | 9 | | See “Data Analysis Strategy” in  Subsection of Methods |
|  |  |  |  | (*b*) Describe any methods used to examine subgroups and interactions | N/A | |  |
|  |  |  |  | (*c*) Explain how missing data were addressed | Table 1  Figure 1 | | See Figure 1 for flowchart  of patients who were  included/excluded.  Patients with missing AUDIT-C  or SOC were excluded  Missing sociodemographic data  was adjusted for in final column of  Table 1 – see Table 1. |
|  |  |  |  | *Cross-sectional study*—If applicable, describe analytical methods taking account of sampling strategy | 5 | | Participants were included among  consecutively admitted medical  inpatients. |
|  |  |  |  | (*e*) Describe any sensitivity analyses | 8 and 21  Table 4  19-20  Table 2 and Table 3 | | To assess our choice of cut-off  AUDIT-C score for women (6) we  Perfomed sensitivity analyses at  Score cut off 5 and 7 for women  We analyzed each potential  cofounder with a univariate logistic  Regression – then included the  Significant variables |
| Results | | | | | | | |
| Participants | | 13* | | (a) Report numbers of individuals at each stage of study—eg numbers potentially eligible, examined for eligibility, confirmed eligible, included in the study, completing follow-up, and analysed | 10 | | As described in flowchart figure 1 |
|  |  |  |  | (b) Give reasons for non-participation at each stage | 10 | |  |
|  |  |  |  | (c) Consider use of a flow diagram | 10 | | Figure 1 |
| Descriptive data | | 14* | | (a) Give characteristics of study participants (eg demographic, clinical, social) and information on exposures and potential confounders | 18 | | Table 1 |
|  |  |  |  | (b) Indicate number of participants with missing data for each variable of interest | 18 | | Table 1 |
|  |  |  |  | (c) *Cohort study*—Summarise follow-up time (eg, average and total amount) |  | |  |
| Outcome data | | 15* | | *Cohort study*—Report numbers of outcome events or summary measures over time |  | |  |
|  |  |  |  | *Case-control study—*Report numbers in each exposure category, or summary measures of exposure |  | |  |
|  |  |  |  | ***Cross-sectional study****—*Report numbers of outcome events or summary measures | 11 | | Stages of change when assessed by  AUDIT-C and PEth as described in  Distribution of stages of change  Under results – and illustrated  in Figure 2 and figure 3 |
| Main results | | 16 | | (*a*) Give unadjusted estimates and, if applicable, confounder-adjusted estimates and their precision (eg, 95% confidence interval). Make clear which confounders were adjusted for and why they were included | 12 and 19-20 | | Tables 2 and 3. |
|  |  |  |  | (*b*) Report category boundaries when continuous variables were categorized | 7 | | Age groups 18-40, 41-67,  68-80, > 80 |
|  |  |  |  | (*c*) If relevant, consider translating estimates of relative risk into absolute risk for a meaningful time period | N/A | |  |
| Other analyses | 17 | | Report other analyses done—eg analyses of subgroups and interactions, and sensitivity analyses | | N/A |  | |
| Discussion | | | | | | | |
| Key results | 18 | | Summarize key results with reference to study objectives | | 9 | Distributions of SOC were significantly  different for increased-risk  consumers (Contemplation OR= 9.63.  CI= 2.2-42 p=0.003 and  Action OR=4.5; CI= 2.16-9.4 p<0.001)  and high-risk consumers (Contemplation  OR=60.5; CI= 13.8-270 p<0.001  and Action OR= 12.6 CI= 5.4-29  p<0.001) when compared to the low risk  consumers.  We found a similar distribution of  the SOC among the three risk groups  when assessed with PEth-concentration  levels” | |
| Limitations | 19 | | Discuss limitations of the study, taking into account sources of potential bias or imprecision. Discuss both direction and magnitude of any potential bias | | 14 | Findings are only in a Norwegian  Population – and should be tested  Internationally. 125 patients excluded  Due to unfinished stages of change quest  Mighty be a selection bias towards the  More motivated patients.  SOC questionnaire was adapted to a  Three-point scale making the results  Difficult to compare to similar studies.  The use of the quick method of allocation  To SOC does not acknowledge the stages  To be mutually exclusive but it probably  Sufficient for the purpose of assessing  the prevalence of SOC. | |
| Interpretation | 20 | | Give a cautious overall interpretation of results considering objectives, limitations, multiplicity of analyses, results from similar studies, and other relevant evidence | | 14-15 | These findings give new insights to  a group of increased-risk drinkers  and potential motivation for alcohol risk-  reduction. We found this group to be  in majority among the patients in action –  a stage associated with drink reduction  it could prove to reduce barriers among  health professionals to screen and advice  this group – as well as inform further  research to assess effects on reduced  alcohol intake. | |
| Generalizability | 21 | | Discuss the generalizability (external validity) of the study results | | 14 | “the study was performed on a large  sample of medical inpatient with a  wide age-range and three different study  sites and should therefore be  representative for a Norwegian  population – however there is a need to  conduct similar research in  other countries to see if these results  are reproducible. | |
| Other information | | |  | | | | |
| Funding | 22 | | Give the source of funding and the role of the funders for the present study and, if applicable, for the original study on which the present article is based | | 1 | **Primary funding:**  The Research Council in Norway (RCN)  Grant/Award Number: 319820. | |

*Give information separately for cases and controls in case-control studies and, if applicable, for exposed and unexposed groups in cohort and cross-sectional studies.

**Note:** An Explanation and Elaboration article discusses each checklist item and gives methodological background and published examples of transparent reporting. The STROBE checklist is best used in conjunction with this article (freely available on the Web sites of PLoS Medicine at http://www.plosmedicine.org/, Annals of Internal Medicine at http://www.annals.org/, and Epidemiology at http://www.epidem.com/). Information on the STROBE Initiative is available at [www.strobe-statement.org](http://www.strobe-statement.org).
